# A network of activists for community-oriented integrated care

**DOI:** 10.1080/17571472.2015.1113713

**Published:** 2015-12-24

**Authors:** Paul Thomas, David Morris

**Affiliations:** ^a^ LJPC

This Issue of LJPC includes papers written by LJPC editors that challenge primary care practitioners and managers in the UK to lead a renaissance of the NHS through the language of *community*-*oriented integrated care* – team-based, locality-based care that has *health* as its focus as well as disease management. This challenge will be made repeatedly throughout 2016 as LJPC papers explore what it is to be healthy at various stages of life, and how primary care can enhance this.

In this Issue, Olivia Martin, a fourth year medical student, felt moved to write about her observation of a GP consultation that was entirely about helping a patient to think through a difficult decision. The role of a GP as facilitator of sense-making is obvious to experienced GPs, because this is how they help people to find health. It lies at the heart of the GP role. Yet it is rarely described either in the popular explanation of what GPs do or, as in the case of this particular student’s training, in content of the professional training curriculum. The challenge is to make this sense-making role widely understood – in training, in strategy and in evaluation.

Peter Toon, retired GP and LJPC editor, picks up the theme of GP as sense-maker through the language of *flourishing narratives* and the lens of virtue ethics. He writes:

most ethical problems which practitioners face are not caused by cutting edge technology or extraordinary situations, but by the normal messy complexities of human life and relationships … [people] are not merely looking for a life which is the longest string of pleasurable experiences possible with the minimum number of unpleasant ones, but for a life which makes sense and has a purpose and a shape.

The challenge is to use consulting styles that help individuals to make sense of the breadth of their diseases, and help groups of people to make sense of their health as whole communities. Primary care practitioners of the future need to be skilled at techniques that help people to help themselves – things, like family conferences, self-help and community development initiatives.

Alison While, Emeritus Professor of Community Nursing and LJPC editor, invites us to practice the health promotion we preach. The healthcare workforce, including primary healthcare staff, exhibits the same health behaviours as the general population – like everyone else we struggle to maintain healthy lifestyles, healthy relationships and healthy organisations. We need to give realistic advice; and in doing so, draw on ourselves as *human beings* and not merely our roles as professionals. We need to be better at putting ourselves into the shoes of others.

In 2016, LJPC will continue the theme of ‘putting one-self into the shoes of others’. When doing this it becomes obvious that different perspectives help to see more of the whole picture of health. Valuing and drawing on multiple perspectives underpin the kind of teamworking that is needed to manage complex conditions and to create integrated care. We will examine what primary care can do to orchestrate collaboration for positive mental health at different stages of life. How can we help children to be mentally healthy – able to play and laugh and adventure in the world? How can we help adults to be good parents and citizens who can be alive in the moment and able to engage optimistically with others? How can the system help doctors and nurses and all others to engage with bigger pictures of health, model healthy behaviours, build healthy teams and design infrastructure for healthy organisations and healthy systems?

In its seven-year history, LJPC has published many outstanding papers written by visionary people. We encourage authors writing about contemporary developments to link their papers to these past papers, and continue their line of thinking about how to address the complex and human factors that make primary care a place with extraordinary potential.

Such scene-setting papers started in the very first Issue of LJPC. Volume One reminded readers of the modern-day implications of the 1978 Alma Ata Declaration. For example:

• John Macdonald’s call to hold to the Alma Ata vision: ‘Whatever language we use to describe it, and however hard it may seem to achieve it, we must not lose sight of an old vision – one which sees health systems as both acknowledging the importance of the social determinants and insists that policy and action are directed upstream as well as downstream’. [1]
• Terry Bamford’s ‘A genuinely patient-centred approach would have the patient and family as part of the team…’ [2]
• Kurt Stange’s call for combined horizontal and vertical integration: ‘As a family physician practicing in the world’s most expensive, lowest value, and perhaps most fragmented health care system (USA), I encourage you to listen to the call to build on the great strength of a system that creates space to work towards the common good’. [3]

And practical ways to apply that vision:

• Clare Gerada’s service for unwell doctors ‘Doctors are in fact at an increased risk of developing depression, burnout and anxiety’. [4]
• Kit Oi Chung and Helen McKendrick’s inner city health centre that was co-designed by the local community: ‘The most important thing we have done is simple and difficult at the same time. It is about listening to people’. [5]
• Anthony Harries experience: ‘My long years in Africa have convinced me about trying to keep things simple and defending the stance of providing a good service to as many people as possible rather than excellence to the few’. [6]
• Indarjit Singh’s wisdom: ‘Different spiritual traditions and secular groups use different techniques to improve the ability to be alive in the moment (it is a very difficult thing to achieve). Primary care practitioners can improve health by helping people to find ways that are meaningful to them to do this’. [7]

Volume One of LJPC also included extracts from John Horder’s autobiography. John, one of the greatest GPs of all time, inspired people the world over to develop broad-visioned, multi-disciplinary primary care. This journal intends to do what it can to further this vision.

LJPC is a network of activists. In the next stage we intend to extend this network by complementing academic writing (submitted for PubMed citation) with large numbers of ‘Landscape’ papers that describe the complexities of health in everyday life – papers like Francesco Carrelli’s review in this Issue of the life of the relationship between Wally Neuzil and Egon Schiele, brought to his attention by an exhibition in the Leopold museum in Vienna.

LJPC also intends to develop Case Studies of *community*-*oriented integrated care* to add richness to the debate about the complexities of making things work, and moderate online discussions to distil principles of success in different contexts.

We wish you a Merry Christmas and a Happy New Year full of resolutions to become active in LJPC.

Paul Thomas paul.thomas7@nhs.net David Morris dmorris1@uclan.ac.uk
